# Efficacy and safety of transesophageal ultrasound-guided patent foramen ovale closure for migraine in adolescents

**DOI:** 10.3389/fped.2023.1296825

**Published:** 2023-11-15

**Authors:** Zeyu Mi, Gang He, Chao Li, Deyu Yang, Xue Liu, Libo Zhao, Hongli Gao, Xing Li, Xiaogang Zhou

**Affiliations:** ^1^ Department of Cardiac Surgery, Yongchuan Hospital of Chongqing Medical University, Chongqing, China; ^2^ Department of Neurology, Yongchuan Hospital of Chongqing Medical University, Chongqing, China; ^3^ Department of Medical Ultrasonics, Yongchuan Hospital of Chongqing Medical University, Chongqing, China

**Keywords:** adolescent, migraine, patent foramen ovale, PFO closure, transesophageal ultrasound

## Abstract

**Objective:**

This retrospective analysis aims to assess the efficacy of transesophageal ultrasound-guided patent foramen ovale (PFO) closure in treating migraine in adolescents and compare the therapeutic outcomes of PFO closure for migraine with and without aura.

**Methods:**

We conducted a retrospective analysis of 86 cases of adolescents (12–20 years old) who underwent PFO closure for migraine at our institution over the past 3 years. The efficacy was evaluated using the visual analogue scale (VAS), headache impact test (HIT)-6, and pediatric migraine disability assessment (PedMIDAS) scores, as well as by assessing the monthly frequency of migraine attacks, duration of each attack, and overall migraine burden. The patients were divided into two groups: an aura group (55 cases) and a non-aura group (31 cases) to investigate difference in therapeutic efficacy between the groups. The effect of residual shunt on migraine burden was assessed.

**Results:**

Among the 86 patients, 46 (54%) experienced complete remission of migraine, while 71 (83%) achieved a >50% reduction in migraine burden during the one-year follow-up period. Patients in the aura group showed more significant improvements in VAS, HIT-6, and PedMIDAS scores, as well as in monthly migraine attack frequency, duration of each attack, and overall migraine burden, than patients in the non-aura group. Moreover, patients with residual shunt did not exhibit statistically significant differences in therapeutic efficacy compared to patients with complete closure.

**Conclusion:**

PFO closure can effectively alleviate migraine symptoms in adolescents with migraine with concomitant PFO. The therapeutic efficacy is particularly pronounced for migraine with aura. Furthermore, minor levels of residual shunt have no effect on the improvement in migraine symptoms.

## 1. Introduction

Migraine ranks first among the causes of disability in adolescents and young adults (1). The incidence of migraine tends to increase from childhood to adolescence and is associated with decreased school attendance and various neurological disorders such as depression, anxiety, epilepsy, and sleep disorders. Migraine has a significant impact on patients and their family members and represents a significant burden on medical and healthcare systems (1, 2).

The foramen ovale, a small passage between the left and right atria normally present during fetal life, serves as a heart structure. After birth, failure of the septum primum and septum secundum to fuse can result in the formation of a patent foramen ovale (PFO). The incidence of PFO in the general population ranges from 20% to 34% (3). A study of 109 adolescents with migraine by McCandless et al. found that the incidence of PFO was 35% (4).

The correlation between PFO and migraine was first proposed by Del Sette et al. in 1998 (5). Subsequently, many observational studies have confirmed that PFO closure is effective in improving the frequency and duration of migraine attacks. In a 2021 pooled analysis of two randomized trials, PRIMA and PRIMARY, Mojadidi et al. concluded that PFO closure significantly reduced the mean monthly migraine days and mean monthly migraine attacks, as well as the number of patients with complete cessation of migraine (6). Despite these findings, the surgical indications for migraine with concomitant PFO remain unclear. In a 2014 study by Menon et al., 153 adolescents with PFO underwent PFO closure, of which 104 underwent the procedure due to migraine, with 97 eventually responding to treatment during the follow-up period. Of these, 68 (70%) reported significant improvement in symptoms, a significantly higher improvement rate than in previous observational studies (7). However, in the Menon et al. study, there was no mention of strict adherence to diagnostic guidelines for migraine in patient selection and diagnosis, and most patients were treated directly by cardiovascular physicians.

In the present study, we evaluated the efficacy of PFO closure in adolescents with migraine and concomitant PFO and retrospectively analyzed the differences in efficacy between the different groups of patients in order to identify patients with migraine and concomitant PFO who are more suitable for surgical treatment.

## 2. Materials and methods

### 2.1. Patient selection

Eighty-six cases of adolescents (12–20 years old) with migraine who underwent PFO closure at Yongchuan Hospital of Chongqing Medical University between Aug 2019 to Jun 2022 were retrospectively analyzed. Patients diagnosed with migraine according to the International Classification of Headache Disorders (ICHD-3) were screened by the Department of Neurology for these migraine-associate PFOs by contrast transcranial Doppler (c-TCD) examination (8). c-TCD-positive patients were then subjected to saline contrast echocardiography to determine the grade of the PFO shunt, and those patients who fulfilled the following criteria were referred to the Center for Cardiovascular Surgery for PFO closure: (a) Failure of previous treatment with one or more anti-migraine therapeutic agents (e.g., treprostinil or ibuprofen) prescribed by a neurologist; (b) The migraine severely affected the patient's daily life, and the patient expressed a strong desire for surgery; (c) The patient showed indications of large shunt based on saline contrast echocardiography, following the four-point right-to-left shunt method. Moreover, we applied specific inclusion and exclusion criteria in selecting patients for inclusion in this study addition to fulfilling the a, b, c above, which are detailed below for clarity.

Patients were included if (1) they were aged 12–20 years; (2) they underwent PFO closure at our Cardiovascular Surgery Center; and (3) they and/or their legal guardians provided written informed consent.

The exclusion criteria were (1) Patients who had migraine with a confirmed cause; (2) Patients who had experienced migraine episodes during less than 6 months; and (3) Patients with an incomplete medical history.

The study was approved by the Institutional Review Board of our hospital (IRB no.:2022-KLSY-101).

### 2.2. Diagnosis and grading of PFO

The diagnosis of PFO was initially confirmed as a positive contrast-transcranial Doppler ultrasonography (c-TCD) result in the Department of Neurology before referral to the Department of Ultrasound Medicine for saline contrast echocardiography. Specifically, a 10-ml syringe containing 1 ml of air and another 10-ml syringe containing 8 ml of saline were obtained. The syringes were connected to a 3-way stopcock, and 1 ml of venous blood was collected and mixed with them. After mixing, the resulting mixture was immediately injected via an elbow vein. The number of air bubbles in the left atrium was observed with the Valsalva maneuver through the four sections of the heart chambers for grading: (1) no shunt: no bubbles observed in the left atrium; (2) small shunt: 1–10 bubbles; (3) medium shunt: 10–30 bubbles; (4) large shunt: over 30 bubbles or a large number of bubbles filling the left atrium.

### 2.3. Transesophageal ultrasound-guided PFO closure procedure

Preoperative testing for each patient included electrocardiography, chest computed tomography, cranial magnetic resonance imaging (MRI), complete blood count, coagulation tests, and migraine score-related questionnaire. After evaluation, transesophageal ultrasound-guided PFO closure was performed. Prior to surgical manipulation, an esophageal ultrasound was used to assess the morphology of the PFO, for example, whether it was (i) a long-tunnel PFO, (ii) accompanied by a Chiari network, and (iii) a low-angle PFO, and to exclude secundum atrial septal defect.

**Figure 1:**
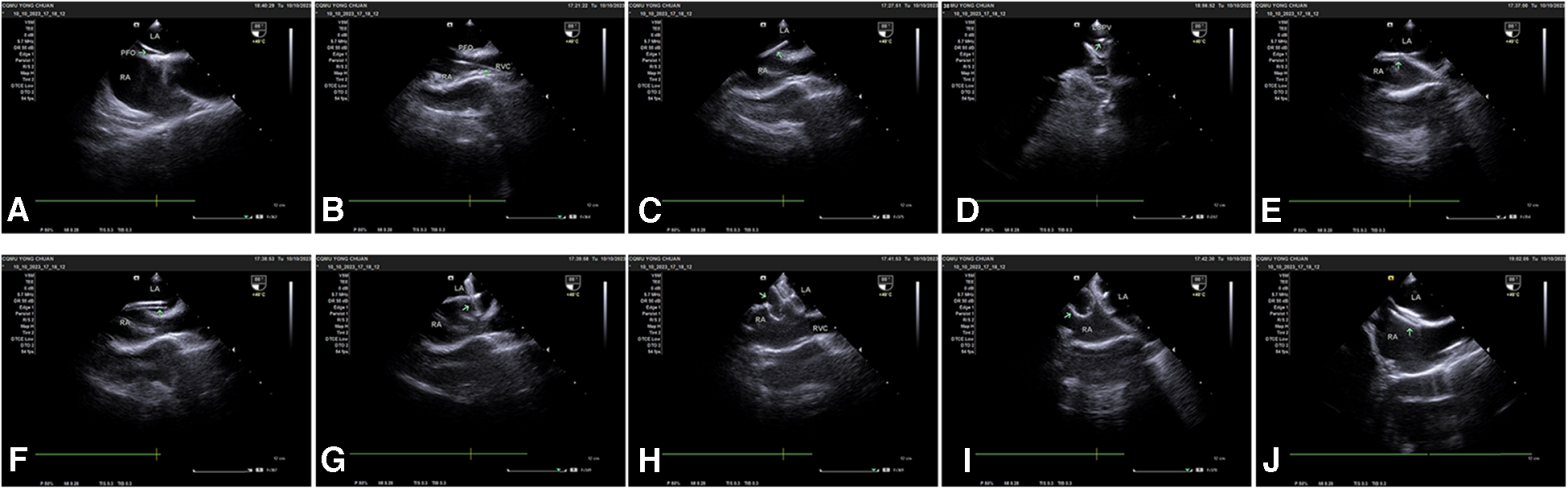
The surgical procedure involved (**A**) transesophageal ultrasound for PFO visualization; (**B**) guidance of the contrast catheter to the interatrial septum with a soft-tip guide wire under transesophageal ultrasound monitoring; (**C**) passage of the guide wire through the PFO; (**D**) placement of the guide wire into the left superior pulmonary vein; (**E**) exchange of the contrast catheter for the occluder delivery sheath; (**F**) withdrawal of the soft-tip guide wire; (**G**) passage of the occluder along the delivery sheath to the left atrium, releasing the left atrial umbrella; (**H**) retraction of the delivery sheath to release the right atrial umbrella; (**I**) traction testing to confirm good fixation of the occluder; (**J**) release of the PFO occluder. All key steps in the diagram are pointed out with green arrows in the diagram.

Details of the transesophageal ultrasound-guided PFO closure surgical procedure are shown in Figure 1. All patients were treated with 100 mg aspirin for six months plus antiplatelet therapy with 75 mg clopidogrel for three months after surgery.

### 2.4. Review data

Data regarding the patient demographics; clinical characteristics; the number of symptoms; preoperative examination and evaluation; surgical procedure details; type of occluder used; complications; postoperative treatment; re-examination results at 6 and 12 months postoperatively; length of hospital stay; and key clinical outcome indicators, including the visual analogue scale (VAS) score, headache impact test (HIT)-6 score, pediatric migraine disability assessment (PedMIDAS) score, frequency of migraine, duration of each migraine attack, and migraine burden, were collected. Migraine burden was defined as the monthly frequency of migraine attacks multiplied by the duration of each attack, it was calculated after pooling data from the final follow-up. During the follow-up, all clinical outcome indicators were evaluated by our neurologists. Improvement of migraine after PFO closure was defined as (1) improvement in migraine-related questionnaire assessment results; (2) complete remission of migraine; and (3) >50% reduction in migraine burden (9).

First, based on retrospective data, we evaluated the efficacy of PFO closure in adolescents with migraine and concomitant PFO. Second, all patients in the study were divided into aura and non-aura groups based on the ICHD-3 diagnostic criteria. The differences in efficacy between the groups were analyzed in accordance with our clinical outcome indicators, and the effect of residual shunt on the improvement of migraine in the patients was evaluated.

### 2.5. Statistical analysis

SPSS 26.0 software was used for statistical analysis. Continuous data are expressed as mean ± standard deviation and were compared using the *t*-test or Mann–Whitney *U* test. Count data are expressed as frequency and/or percentage and were compared using the *χ*^2^ test. Differences with *p* ≤ 0.05 were considered statistically significant.

## 3. Results

Eighty-six patients met the inclusion criteria. There were 22 males (23%) and 66 females (77%). The ages of the patients ranged from 12 to 20 years, and the mean age was 15.95 ± 2.17 years. The mean duration of the disease was 2.43 ± 2.25 years. There were 55 (64%) cases of migraine with aura and 31 (36%) of migraine without aura. We also ascertained other possible causes of headache, including four cases of a right-to-left shunt at the lung level, 12 cases of sinusitis, and 12 cases of transient ischemic attacks (TIA). The primary clinical outcome indicators, which included the VAS score, HIT-6 score, PedMIDAS score, monthly frequency of migraine attacks, duration of migraine attacks, and migraine burden, were 7.63 ± 0.88, 66.94 ± 5.08, 49.73 ± 7.47, 7.28 ± 2.65, and 12.31 ± 3.46 respectively. The baseline characteristics of the study cohort are presented in Table 1.

**Table 1:** Baseline characteristics of the adolescents with migraine combined with PFO.

Variables	*n* (%)
Total	86
Female	66 (77%)
Age (years)	15.95 ± 2.17
Course of disease (years)	2.43 ± 2.25
Combination of symptoms	
Dizziness	46 (53%)
Vision symptoms	17 (20%)
Chest symptoms	36 (42%)
Combined brain imaging microemboli embolism	5 (6%)
Combination of other possible causes of headache	
Lung level shunt	4 (5%)
TIA	12 (14%)
Rhinosinusitis	12 (14%)
History of depression	12 (14%)
Migraine characteristics	
MA	55 (64%)
MOA	31 (36%)
VAS	7.63 ± 0.88
HIT-6	66.94 ± 5.08
PedMIDAS	49.73 ± 7.47
Frequency (per month)	7.28 ± 2.65
Duration (hours)	12.31 ± 3.46
Migraine burden	91.87 ± 44.61

PFO, patent foramen ovale; TIA, transient ischemic attacks; VAS, Visual Analogue Scale; HIT-6, Headache Impact Test; PedMIDAS, Pediatric Migraine Disability Assessment; MA, migraine with aura; MOA, migraine without aura.

### 3.1. Outcomes of the transesophageal ultrasound-guided PFO closure

All patients were admitted to our neurology department and were transferred to the cardiovascular surgery department for PFO closure. The entire procedure was performed under transesophageal ultrasound guidance, and occluders were implanted with a 100% success rate. The Abbott PFO occluder was used in 67 cases (78%), and the Starway Medical (Beijing, China) PFO occluder was used in 19 (22%). The average surgical duration was 25.72 ± 11.28 min, and the average length of hospital stay was 3.89 ± 1.67 days (Table 2).

**Table 2:** Results of the PFO closure procedure.

	*n* (%)
Total	86
Device used	
Amplatzer	67 (78%)
Starway Medical	19 (22%)
Complications	
Pericardial effusion	1 (1%)
Arrhythmia	4 (5%)
Duration of surgery (min)	25.72 ± 11.28
Length of hospitalization (day)	3.89 ± 1.67
Residual shunt	26 (30%)
Large	4
Medium	11
Small	11

PFO, patent foramen ovale.

Postoperative adverse events included one case of delayed small pericardial effusion and four cases of arrhythmia, all of which were cases of paroxysmal atrial fibrillation that recovered completely with symptomatic treatment. Another patient experienced palpitations after strenuous exercise 3 months postoperatively that did not resolve 12 months postoperatively; however, the patient did not experience significant discomfort with daily activities. There were no cases of adverse events such as thromboembolism, occluder dislodgement, hemorrhagic events, or other cardiac injuries at the 12-month follow-up. Overall, the adverse event rate was low.

### 3.2. Improvement in migraine symptoms

Most patients had varying degrees of improvement in migraine symptoms following PFO closure. By the 12-month follow-up, complete remission was achieved in 46 patients (54%), and the migraine burden was reduced by >50% in 71 patients (83%). Twelve patients (14%) had no significant improvement after PFO closure. However, there were no cases of worsened migraine after the PFO closure. Data regarding the VAS score; HIT-6 score; PedMIDAS score; frequency of migraine attacks; duration of migraine attacks; and migraine burden at baseline, 6 months postoperatively, and 12 months postoperatively are presented in Table 3. The primary clinical outcome indicators decreased significantly from baseline to 12 months postoperatively (all *p* < 0.05, Table 3).

**Table 3:** Evaluation of the clinical efficacy of the PFO closure procedure in adolescents with migraine.

	M0	M6	M12
VAS	7.63 ± 0.87	2.56 ± 2.85	2.12 ± 2.65
HIT-6	66.94 ± 4.80	46.28 ± 12.25	43.99 ± 10.85
PedMIDAS	49.73 ± 7.52	12.40 ± 15.63	8.60 ± 15.38
Frequency	7.35 ± 2.60	2.23 ± 2.90	1.60 ± 2.72
Duration	12.34 ± 3.44	4.05 ± 4.88	3.27 ± 4.73
Migraine burden	91.87 ± 44.62	20.73 ± 34.13	15.48 ± 34.42

VAS, Visual Analogue Scale; HIT-6, Headache Impact Test; PedMIDSA, Pediatric Migraine Disability Assessment; M0, Baseline; M6, at 6 months postoperatively; M12, at 12 months postoperatively; PFO, patent foramen ovale.

### 3.3. Improvement in migraine with and without aura

**Figure 2:**
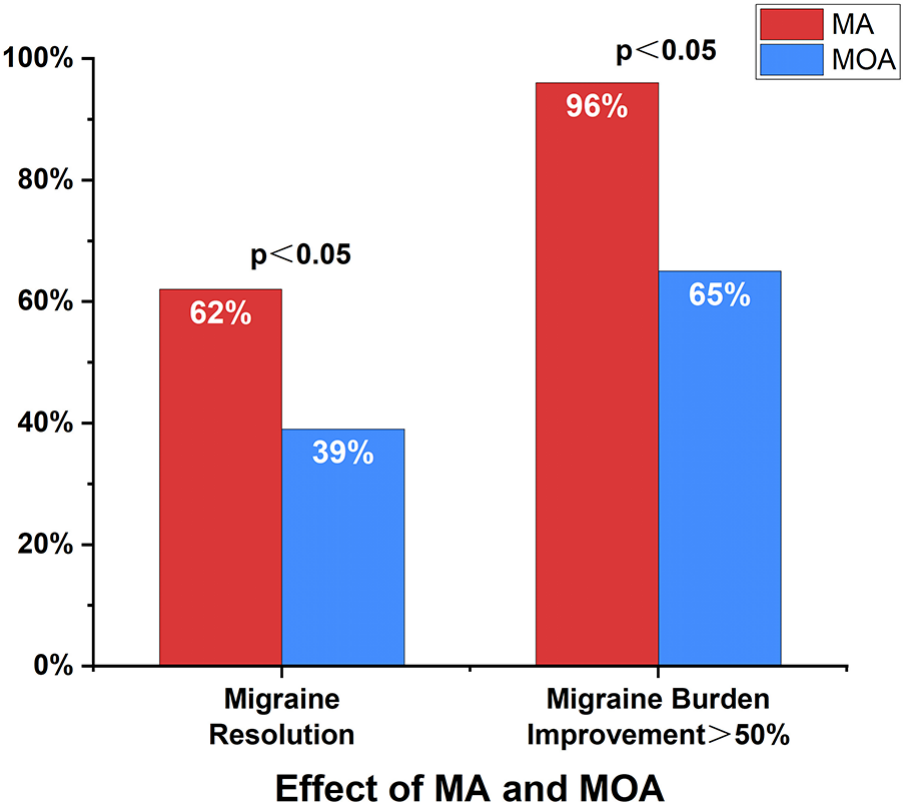
Improvement in migraine with and without aura. At the 12-month follow-up, a total of 46 cases (54%) had complete cessation of migraine, and 71 cases (83%) had >50% reduction in migraine burden. Complete cessation of migraine and >50% reduction in migraine burden were more likely to occur in the aura group. MA, migraine with aura; MOA, migraine without aura.

**Figure 3:**
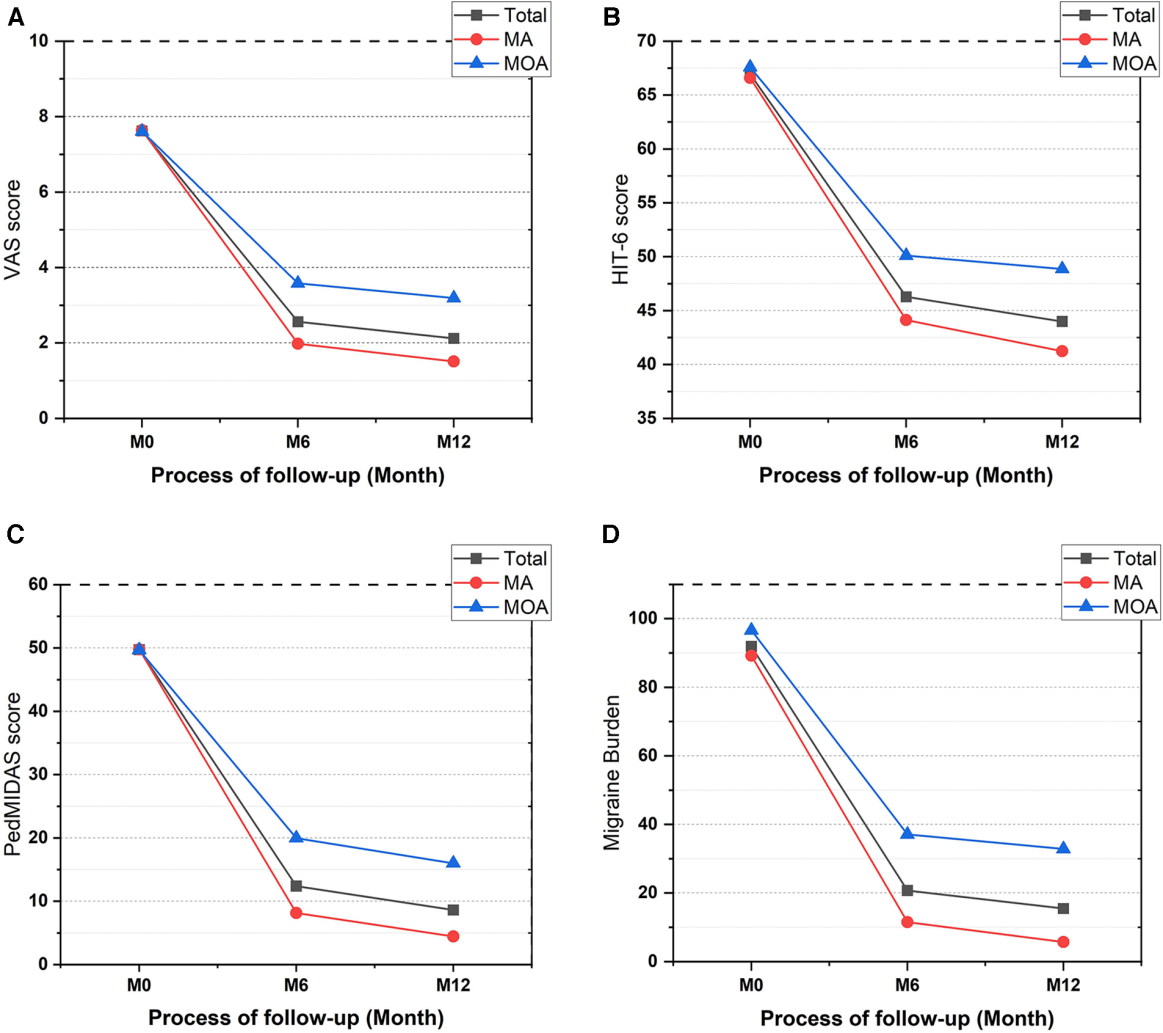
Improvement of clinical outcome indicators in migraine with and without aura. (**A**) Improvement of VAS, (**B**) improvement of HIT-6, (**C**) improvement of PedMIDAS, (**D**) improvement of migraine burden. VAS, Visual Analogue Scale; HIT-6, Headache Impact Test; PedMIDAS, Pediatric Migraine Disability Assessment; MA, migraine with aura; MOA, migraine without aura; M0, baseline; M6, at 6 months postoperatively; M12, at 12 months postoperatively.

Of the 86 patients, there were 55 cases of migraine with aura (64%) and 31 cases of migraine without aura (36%). There were no differences regarding gender, age, or duration of disease between the two groups. At the 12-month follow-up, 34 patients in the aura group (62%) and 12 in the non-aura group (39%) had complete cessation of migraine. Fifty-three patients in the aura group (96%) and 20 in the non-aura group (65%) had >50% reduction in migraine burden (Figure 2). The differences were statistically significant. The aura and non-aura groups also exhibited significant differences in VAS scores, HIT-6 scores, PedMIDAS scores, and migraine burden, with more significant decreases in the aura group than in the non-aura group (Table 4 and Figure 3).

**Table 4:** Intergroup differences regarding MA and MOA.

Projects	Groups	Intra-group differences			Intergroup differences	
		M0	M12	*p*-Value	M12 Project change	*p*-Value
VAS	MA	7.64 ± 0.97	1.51 ± 2.27	<0.05	−1.68 ± 0.62	0.009
	MOA	7.61 ± 0.67	3.19 ± 2.97	<0.05		
HIT-6	MA	66.58 ± 4.58	41.24 ± 8.60	<0.05	−7.63 ± 2.56	0.005
	MOA	67.58 ± 5.18	48.87 ± 12.73	<0.05		
PedMIDAS	MA	49.76 ± 7.64	4.44 ± 9.82	<0.05	−11.56 ± 3.86	0.005
	MOA	49.68 ± 7.42	16.00 ± 20.21	<0.05		
Migraine burden	MA	89.21 ± 45.42	5.69 ± 16.05	<0.05	−27.15 ± 9.07	0.005
	MOA	96.58 ± 43.48	32.84 ± 49.06	<0.05		

VAS, Visual Analogue Scale; HIT-6, Headache Impact Test; PedMIDAS, Pediatric Migraine Disability Assessment; MA, migraine with aura; MOA, migraine without aura; M0, Baseline; M12, at 12 months postoperative; PFO, patent foramen ovale.

### 3.4. Migraine outcomes with different PFO closure outcomes

Among all patients, there were 26 cases of residual shunt (30%), including four cases of large residual shunt, 11 cases of medium residual shunt, and 11 cases of small residual shunt. Among patients with residual shunt, 19 cases were treated with the Abbott occluder, and seven were treated with the Starway occluder. The difference in the incidence of residual shunting between the two occluders was not statistically significant. Furthermore, there were no statistically significant differences in primary clinical outcome indicators between the patients with complete closure of the foramen ovale and those with a residual right-to-left shunt.

### 3.5. Outcome of syndromes associated with PFO

Some of the concomitant symptoms of migraine and other disorders that may contribute to migraine were also retrospectively analyzed in this study cohort. Among them, there were 46, 17, and 36 cases of concomitant vertigo symptoms, visual symptoms, and chest symptoms, respectively. These symptoms also exhibited varying degrees of improvement over the follow-up period. We observed that of the 12 patients with concomitant TIA, nine did not have another TIA after PFO closure. Of the 12 patients who did no significant improvement of migraine, seven had sinusitis, as indicated by the cranial MRI prior to admission. Moreover, in the four patients with a concomitant right-to-left shunt at the lung level, two had complete cessation of migraine, and two had >50% reduction in migraine burden.

## 4. Discussion

Migraine is a disabling disease that severely affects the quality of life of both adults and adolescents (1). It is one of the most common primary causes of headaches in children and adolescents. It affects physical and mental health, school attendance, and family and social activities, leading to a reduced quality of life and causing severe disability (10). A large epidemiological survey in the United States showed that the prevalence of migraine at the age of 15 years ranged from 8% to 23%. The prevalence is slightly higher in boys compared to girls before puberty, and subsequently becomes higher in girls compared to boys after the age of 11 years (11). In addition, most migraines in adults originate during childhood and adolescence, and early intervention in adolescence can significantly improve quality of life and reduce disability in later life (12). Numerous studies in recent years have confirmed that PFO and migraine are highly co-morbid, and the prevalence of PFO in particular is two times higher in migraine patients with aura, relative to the general population (13).

While there has recently some progress in terms of acute pharmacological prophylaxis, behavioral therapy, and neuromodulation devices, it is important to note that the impact of migraine on adolescents remains a significant challenge. To date, there is no definitive treatment for migraine in adolescents (14–16). In recent years, an increasing number of observational studies have described the correlation between migraine and PFO in adults and demonstrated that PFO occlusion is effective in reducing the frequency and duration of migraine, especially in migraine with aura (9, 17–24). Although the MIST, PREMIUM, and PRIMA randomized controlled trials did not meet their primary endpoints, the PREMIUM and PRIMA trials confirmed the efficacy of PFO closure in reducing the frequency and duration of migraine. Although the MIST trial concluded that PFO closure did not have a beneficial effect on migraine, a shortcoming of the trial was the short follow-up period of 6 months. The mean age of the population with PFO in the three RCT trials was 44.3 ± 10.6, 42.8 ± 10.3, and 44.1 ± 10.7 years, respectively. In most observational studies, the mean age of the population with PFO was in the low to mid-40 s, and no studies to date have focused exclusively on the treatment of migraine in adolescents with PFO closure.

We conducted a retrospective study involving 86 adolescents with migraine and concomitant PFO admitted to our center in the last 3 years. Standard diagnostic and status assessments for migraine were performed before and after the operation to observe the efficacy of PFO closure in this cohort of patients and compare between groups, with the goal of identifying adolescent patients with migraine and concomitant PFO who are more suitable for PFO closure surgery. In addition, our findings were compared with those from previous studies in adults.

Our results showed that at the 12-month follow-up, 46 patients (54%) had complete cessation of migraine and 71 (83%) had >50% reduction in migraine burden, consistent with the results of a 2022 study by Qi et al. in which 134 patients with migraine were followed up for 12 months. In that study, 54 patients (40%) had complete remission of migraine, and 102 (76%) had >50% reduction in migraine attacks (23). Our evaluation metrics included the VAS score, HIT-6 score, PedMIDAS score, monthly frequency of migraine attacks, duration of each attack, and migraine burden. In all patients, the primary outcome indicators were significantly lower at the 12-month follow-up after percutaneous PFO closure compared to baseline. In a study by Rigatelli et al., the MIDAS score was used as the primary outcome indicator. During the follow-up period, the MIDAS score decreased from 35.8 ± 4.7 before PFO closure to 8.3 ± 7.8 after closure (19). In our study, the PedMIDAS decreased from 49.73 ± 7.52 before PFO closure to 8.60 ± 15.38 after closure in all patients, similar to previous studies. However, the primary clinical outcome indicators in our study were higher than previous adult studies at baseline (19–23). This may be because adolescents have a lower tolerance for migraine than adults.

Previous studies showed that the incidence of PFO was high in patients with migraine with aura in both adult and adolescent populations and might be more closely associated with migraine (4, 25). In addition, many studies have demonstrated that PFO closure is more effective for migraine with aura (6, 26). Mojadidi et al. (6) pooled two previous randomized trials and found that patients with migraine with aura who underwent PFO closure exhibited a significant reduction in monthly migraine days compared to controls (−3.2 ± 4.8 days vs. −1.8 ± 4.4 days, *p* = 0.03), as well as a significant difference in the number of patients with complete cessation of migraine (11 cases vs. 1 case, *p* = 0.002). In contrast, patients with migraine without aura did not have a significant reduction in the number of migraine days after undergoing PFO closure (−2.8 ± 3.4 days vs. −2.2 ± 4.0 days, *p* = 0.53); moreover, there was no statistically significant difference between patients with complete cessation of migraine and controls (5 cases vs. 0 cases, *p* = 0.16). The study also mentioned that patients with migraines with frequent auras had better outcomes than those with infrequent auras. In the present study, patients in the aura group had a higher probability of complete cessation of migraine after PFO closure than those in the non-aura group (62% vs. 39%, *p* < 0.05). Similar results were observed for a >50% reduction in migraine burden (96% vs. 65%, *p* < 0.05). Moreover, it was found that PFO closure was more effective for migraine with aura than for migraine without aura in the treatment of adolescents with migraine with concomitant PFO, similar to studies in adults.

With respect to previous observational studies, PFO closure for the treatment of migraine was confirmed to have some efficacy; however, there have been few studies on the effect of postoperative residual shunt on migraine burden. In a study by Matsumura et al., the residual shunt rates with five occluders were compared over a 12-month follow-up period, and the rate of the residual shunt for PFO occluders was 14% (27). In a study by Ben-Assa et al., 29 of 110 patients (26%) had varying degrees of residual shunt 6 months postoperatively, and migraine improvement was more pronounced in patients with complete closure than in those with residual shunt (91% vs. 76%, *p* = 0.032) (9). In two other studies, the extent of the residual shunt at 1 day and 1 month postoperatively were assessed, but the migraine assessment questionnaires were collected at 6 and 12 months postoperatively. Occluders require a long-term endothelialization process after implantation in the heart, a process that can take months or even years; therefore, the results obtained by the researchers at the end of the follow-up period in these two studies may not have reflected the effect of residual shunt on migraine burden. In a recent study with a 10-year follow-up period, the presence of residual shunt was observed in 4.7% of 441 patients after the operation; nonetheless, the percentage of patients with residual shunt decreased over the overall follow-up period. At 9.2 years of follow-up, only three patients had moderate to severe residual shunt, an incidence of less than 1% (28). In the present study, 26 patients (30%) had residual shunt 12 months postoperatively. Moreover, there were four cases of a large residual shunt, 11 of a medium residual shunt, and 11 of a small residual shunt. There was no significant difference in the improvement in migraine symptoms between patients with complete occlusion and those with a residual shunt. Wang et al. found that a large amount of right to left shunt (RLS) was strongly associated with migraine, while the proportion of mild and moderate RLS in the migraine population did not differ significantly from that of the general population (29). The concept of “effective closure” in stroke prevention studies is defined as little or no right-to-left shunt, which is sufficient to prevent strokes associated with PFO (30). If this same concept is applied to the treatment of migraine with PFO, and if small and moderate residual shunts are considered as effective occlusion, then 82 of the 86 patients in this study (95%) achieved effective occlusion 12 months postoperatively. This may be attributed to the absence of significant differences in migraine treatment outcomes between the two groups of patients with complete occlusion and those with a residual shunt.

In addition, among the follow-up patients, there were 12 with concomitant TIA, of which 9 did not experience another TIA after PFO closure. This is consistent with the results in stroke prevention studies regarding PFO (28). In the present study, we also found that seven out of the 12 patients with no significant improvement of migraine had concomitant sinusitis at baseline, which could potentially account for their failure to achieve remission of migraine after PFO closure (8). Nonetheless, we did not re-evaluate this group of patients for sinusitis during the 12-month follow-up period.

The search for the mechanism of migraine due to PFO has been continuous, and its pathogenesis has been hypothesized to be related to paradoxical embolism, vasoactive substances, hypoxia, and genetics. In addition, some evidence from neurological imaging has suggested a correlation between PFO and migraine. However, the mechanism of migraine development due to PFO remains unclear (31–34). Paradoxical embolism is the mechanism of PFO-induced migraine that is currently more accepted. Paradoxical embolism refers to the bypassing of the pulmonary circulation by emboli originating from the right atrium or venous system through the unclosed PFO to reach the corporeal circulation directly, causing arterial embolism. Microemboli from the venous system lead to insufficient local cerebral tissue perfusion due to the lack of filtration by lung tissue, resulting in transient cerebral hypoxia, which induces cortical spreading depression (CSD) and triggers a migraine headache. CSD is thought to be an inhibitory band of electrical activity originating in the occipital lobe that is associated with the development of aura during migraines, which corresponds to the neurological theory on migraine pathogenesis (35–37). Moreover, because paradoxical embolism is more likely to occur in the posterior circulation, hypoperfusion of the relevant sites is prone to cause visual aura, which is in line with the current study, supporting the idea that MA is more common in patients with combined PFO (38). It was found that only MA patients with larger PFOs showed significant spectral power changes after intravenous injection of air microemboli. This suggests that larger PFOs may allow more microemboli to enter the circulation, thereby triggering migraine through the paradoxical embolism mechanism (39, 40). In our study, all patients were heavily shunted, which may have been one of the reasons why the migraine burden was reduced by >50% in 71 patients (83%).

All patients were treated with aspirin for six months and with clopidogrel for three months after PFO closure. Aspirin was selected as a general prophylactic drug against migraine, and clopidogrel as an effective supplemental prophylactic drug for migraine combined with PFO. Therefore, either or both may have biased our observations (23). However, our follow-up period was 12 months long, and all patients in our study had used one or more antimigraine therapeutic agents which had failed or had a poor outcome. Therefore, although we could not exclude the effect of antiplatelet drugs on migraine in our patients, the results of the present observational study corresponding to the twelfth postoperative month of follow-up still suggest that PFO closure has some efficacy in the prevention of adolescent migraine.

Migraine and changes in hormone levels are closely related, mainly in terms of the effect of plasma estrogen levels, with decreases triggering migraine attacks without aura, while higher estrogen levels appear to have a protective effect. Migraine may spontaneously improve when adolescent patients achieve stable hormone levels (41). The effect of fluctuating hormone levels on migraine improvement could not be ruled out in our observational study, and therefore it would be important for future investigators to determine how this should be taken into account when PFO closure is performed in cases of adolescent migraine combined with PFO. In addition, we intend to perform further studies with long-term follow-up periods as part of our future work.

Furthermore, transesophageal ultrasound is the gold standard for the diagnosis and evaluation of PFO. PFO closure under transesophageal ultrasound guidance allows the morphology of the PFO to be evaluated prior to occluder implantation and the selection of the size of the occluder. It also enables real-time monitoring of the position of the occluder during implantation, assessment of any residual shunt, visualization of the relationship between the implanted occluder and the surrounding structures, and other advantages. It also allows for occlude recovery if implantation was inappropriate. All 86 patients in the present study were implanted with an occluder under transesophageal ultrasound guidance with a 100% success rate and a low number of postoperative adverse events, which was within acceptable limits. This demonstrates that PFO closure in adolescents performed under transesophageal ultrasound monitoring and guidance is safe and effective. The safety of the procedure is based on maximizing the success rate of interventional occlusion. Furthermore, in contrast to implantation under radiographic guidance, transesophageal ultrasound guidance reduces the amount of radiation absorbed by both the patient and the physician, and avoids potential further exacerbation of renal impairment due to the use of contrast agents in patients with partial renal impairment, thus providing socioeconomic benefits.

### 4.1. Study limitations

First, this was a retrospective study, and all primary clinical outcome indicators were obtained from patient recall at baseline and 6 and 12 months postoperatively without rigorous completion of headache diary cards. These may have led to recall bias.

Second, no medication control group was established in this study, and biased results due to placebo effects cannot be excluded. In addition, all patients were treated with aspirin for 6 months and clopidogrel for 3 months after the operation; therefore, the effect of antiplatelet drugs on migraine cannot be excluded.

## 5. Conclusions

PFO closure appears to be effective in improving migraine symptoms in adolescents with migraine and concomitant PFO and is more effective in cases of migraine with aura. Furthermore, minor residual shunts do not appear to impact the improvement of migraine symptoms. Given that there are still no clear treatment options for migraine in adolescents, PFO closure may represent a novel option for the treatment of migraine in adolescents. However, further research on the population for which PFO closure is indicated for the treatment of migraine is required.

## Data Availability

The original contributions presented in the study are included in the article/Supplementary Material, further inquiries can be directed to the corresponding author.
